# Age-related decline in the taurine content of the skin in rodents

**DOI:** 10.1007/s00726-021-02956-2

**Published:** 2021-02-20

**Authors:** Tomohisa Yoshimura, Yuki Inokuchi, Chikako Mutou, Takanobu Sakurai, Tohru Nagahama, Shigeru Murakami

**Affiliations:** 1 R&D Laboratories, Self-Medication, Taisho Pharmaceutical Co. Ltd, 403, Yoshino-cho 1-chome, Kita-ku, Saitama-shi, Saitama, 331-9530 Japan; 2 Drug Safety and Pharmacokinetics Laboratories, Taisho Pharmaceutical Co. Ltd, 403, Yoshino-cho 1-chome, Kita-ku, Saitama-shi, Saitama, 331-9530 Japan; 3 Department of Bioscience and Biotechnology, Fukui Prefectural University, 4-1-1 Kenjojima, Matsuoka, Eiheiji-Town, Fukui, 910-1195 Japan

**Keywords:** Taurine, Skin, Aging, Epidermis

## Abstract

Taurine, a sulfur-containing amino acid, occurs at high concentrations in the skin, and plays a role in maintaining the homeostasis of the skin. We investigated the effects of aging on the content and localization of taurine in the skin of mice and rats. Taurine was extracted from the skin samples of hairless mice and Sprague Dawley rats, and the taurine content of the skin was determined by high-performance liquid chromatography (HPLC). The results of the investigation revealed that the taurine content in both the dermis and epidermis of hairless mice declined significantly with age. Similar age-related decline in the skin taurine content was also observed in rats. In contrast, the taurine content in the sole remained unchanged with age. An immunohistochemical analysis also revealed a decreased skin taurine content in aged animals compared with younger animals, although no significant differences in the localization of taurine were observed between the two age groups. Supplementation of the drinking water of aged mice with 3% (w/v) taurine for 4 weeks increased the taurine content of the epidermis, but not the dermis. The present study showed for the first time that the taurine content of the skin decreased with age in mice and rats, which may be related to the impairment of the skin homeostasis observed with aging. The decreased taurine content of the epidermis in aged animals was able to be rescued by taurine supplementation.

## Introduction

Taurine (2-aminomethylsulfonic acid) is a sulfur-containing amino acid that occurs in abundance in mammalian tissues. Taurine is widely recognized as being involved in various biological and physiological processes in the body, through exerting basic effects, such as osmoregulatory, antioxidant, anti-inflammatory, membrane stabilizing, and Ca^2+^ mobilizing activities (Huxtable 1992; Schaffer et al. 2000, 2003). It is thought to be an important substance for maintaining whole-body homeostasis. In fact, knockout of the taurine transporter to induce taurine deficiency in mice resulted in many abnormalities, including disorders of glucose and lipid metabolism (Ito et al. 2015), and dysfunctions of the skeletal muscle (Ito et al. 2014), heart (Ito et al. 2008), liver (Warskulat et al. 2006), and central nervous system (Sergeeva et al. 2007). Therefore, decline in the tissue content of taurine may cause impairment of organ functions.

The main function of the skin is to protect the body against mechanical traumas, pathogens, radiation, and excessive water loss. The skin consists of three layers: the epidermis, dermis, and subcutaneous tissue. The epidermis is composed, from the surface, of the stratum corneum, granular layer, spinous layer, and basal layer, and is responsible for the moisturizing and barrier functions of the skin (Madison 2003). It has been shown that taurine occurs predominantly in the epidermis, especially in the epidermal granular layer (Lobo et al. 2001). Several studies have revealed that taurine plays significant roles in moisture retention and UV protection of the skin, through exerting osmoregulatory, and anti-inflammatory actions (Janeke et al. 2003; Anderheggen et al. 2006; Rockel et al. 2007). Thus, taurine is thought to be essential for maintaining the normal skin functions. With advancing age, both epidermal homeostasis and the skin barrier function are impaired (Al-Nuaimi et al. 2014). For example, a decrease in the intracellular lipid content in aged skin results in increased transepidermal water loss (TEWL) and increased susceptibility to skin irritants and xerosis. Although the beneficial effect of taurine in maintaining the skin barrier function has been confirmed, changes in the skin taurine content with aging have not yet been well studied. In the present study, we conducted animal experiments using mice and rats to examine whether the taurine content of the skin decreases with aging. In addition, we assessed the effect of taurine supplementation of drinking water on the taurine content of the skin.

## Materials and methods

### Animals

Male hairless mice (Hr-1) and male Sprague Dawley rats were obtained from Japan SLC (Hamamatsu, Japan) and housed in an SPF, temperature-, and humidity-controlled room maintained under a 12-h light–dark cycle. The animals had free accesses to a commercial diet (MF, Oriental Yeast Co., LTD.) and water throughout the experimental period. Each experiment was performed using 6- to 39-week-old animals. The experimental protocol was approved by the Taisho Pharmaceutical Company Animal Care Committee.

### Taurine supplementation of drinking water

Taurine (Wako Pure Chemical, Osaka, Japan) was dissolved in drinking water at a concentration of 3% (w/v) and given ad libitum for 4 weeks to 35-week-old mice. The dose and duration of oral taurine supplementation were determined by referring to the previous studies (Ideishi et al. 1994; Dawson et al. 2002).

### Measurement of the skin taurine content

The mice and rats were sacrificed under anesthesia for skin tissue collection. The collected skin samples were fixed in a petri dish, wrapped in plastic wrap, and floated in a warm bath at 60 °C for 2 min. They were further floated on ice water for 2 min, and the epidermis, dermis, and sole skin were peeled. Each tissue sample was measured and stored at − 80 °C until analysis. For the analysis, each skin sample was thawed and 10 volumes of methanol was added, followed by homogenization by sonication for 1 min (VCX-750, Sonics & Materials Inc, Newtown, CT, USA) and centrifugation at 800×*g* for 15 min. The resultant supernatants were filtered through a 0.22-μm filter and further diluted with methanol to quantify the taurine content of the skin by online high-performance liquid chromatography (HPLC) using the precolumn fluorescence derivatization reagent, *o*-phthalaldehyde (OPA) (MP Biomedicals, Santa Ana, CA, USA). The HPLC experiments were performed on a system equipped with NANOSPACE SI-1, Guard column (CAPCELL C18 MG, S-5, 10 × 2.0 mm I.D) and Analytical column (CAPCELL PAK C18 MG, S-5, 150 × 2.0 mm I.D) (Shiseido, Tokyo, Japan). The chromatographic conditions were as follows: the mobile phase was a mixture of methanol: 0.01 M PBS (pH 6.8) at the ratio of 2:3 (v/v). The flow rate was 180 μL/min. The temperature column oven was adjusted to 30 °C. The detection wavelengths were set for fluorescence detection (Ex. 340 nm, Em 450 nm).

### Immunohistochemical analysis of the skin

Skin samples of 6- and 24-week-old mice were collected from the dorsal skin of the animals. The samples were immersion-fixed in 4% paraformaldehyde and 0.5% glutaraldehyde in 0.1 M phosphate buffer and embedded in paraffin. For immunostaining of taurine, the dewaxed paraffin sections were pre-incubated with 10 µg/mL protease K (03,115,828,001, Roche Diagnostics K.K., Japan) at 37ºC for antigen retrieval, blocked with 1% bovine serum albumin and Avidin/Biotin Blocking kit (SP-2001, Vector Laboratories, Burlingame, CA, USA). Then, the sections were incubated with anti-taurine antibody (2.5 µg/mL, Y052723, Applied Biological Materials Inc. Richmond, Canada) overnight at 4 ºC. Then, the secondary antibody, biotin-conjugated goat antirabbit IgG (BA-1000, 10 μg/mL, Vector Laboratories), was applied for 1 h at room temperature. The slides were exposed to 0.3% H_2_O_2_ for 30 min to quench endogenous peroxidase. The sections were incubated using the ABC Elite Standard kit (PK-6100, Vector Laboratories, Burlingame, CA, USA). Finally, the peroxidase activity was visualized with the AEC Staining kit (AEC-101, Sigma-Aldrich, St. Louis, MO, USA). Negative controls were incubated with normal rabbit IgG (20304, 2.5 µg⁄mL, Imgenex, San Diego, CA, USA) instead of with the primary antibodies.

### Statistical analysis

All values are expressed as the means ± S.E. The results were statistically analyzed by one-way ANOVA, followed by Dunnett’s test, or Student’s *t* test, using the SAS preclinical package software, version 5.0 (SAS Institute Japan Co. Ltd., Tokyo, Japan).

## Results

### Effect of aging on the skin taurine content

**Fig. 1:**
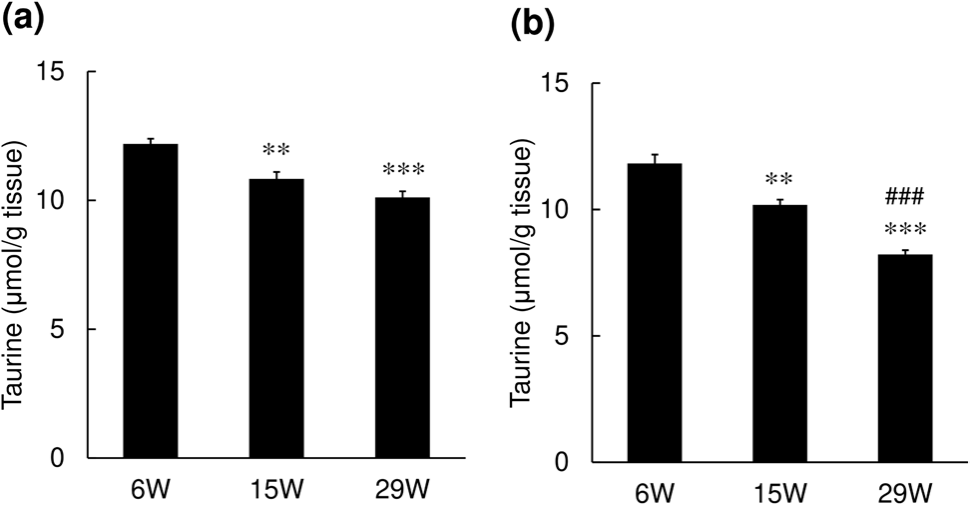
Changes in the taurine content of the skin in mice. Skin samples were excised from the backs of hairless mice, and the taurine content was determined in the epidermis (**a**) and dermis (**b**) by HPLC. Values represent the mean ± S.E. of 7–10 animals. The values were significantly different from those in 6-week-old mice (**p < 0.01, ***p < 0.001) and those in 15-week-old mice (^###^*p* < 0.001), determined by ANOVA (Dunnett’s test)

**Fig. 2:**
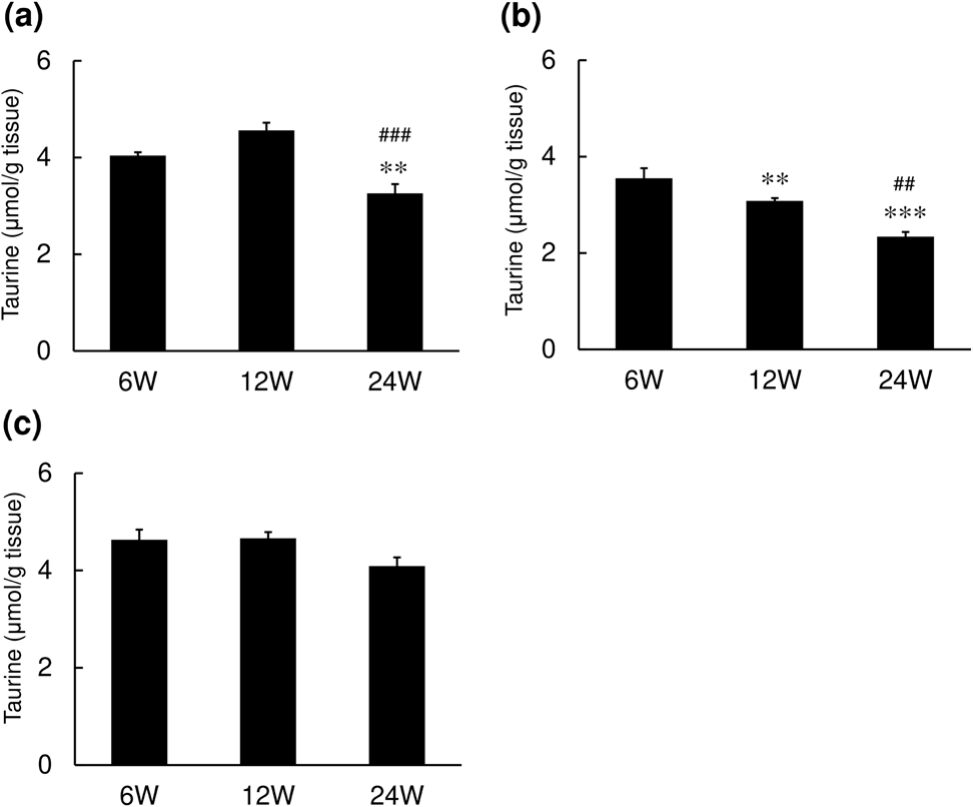
Changes in the taurine content of the skin in rats. Skin samples were excised from the backs of SD rats, and the taurine content was determined in the epidermis (**a**), dermis (**b**) and sole (**c**) by HPLC. Values represent the mean ± SE of 7–10 animals. The values were significantly different from those in 6-week-old mice (**p < 0.01, ***p < 0.001) and those in 12-week-old mice (^##^*p* < 0.01, ^###^*p* < 0.001), determined by ANOVA (Dunnett’s test)

We collected the skin from hairless mice and rats, and compared the taurine content of the skin in young animals and their aged counterparts. The taurine contents in both the epidermis (Fig. 1a) and dermis (Fig. 1b) were significantly decreased in the 15- and 29-week-old mice as compared to the 6-week-old mice. Similar age-related decline was also observed in the skin of the rats. The taurine content of the epidermis in the 24-week-old rats was significantly lower than that in 6- and 12-week-old rats (Fig. 2a). The taurine content of the dermis also decreased with age (Fig. 2b). In contrast, the taurine content in the sole remained unchanged with age (Fig. 2c).

### Immunohistochemical analysis

**Fig. 3:**
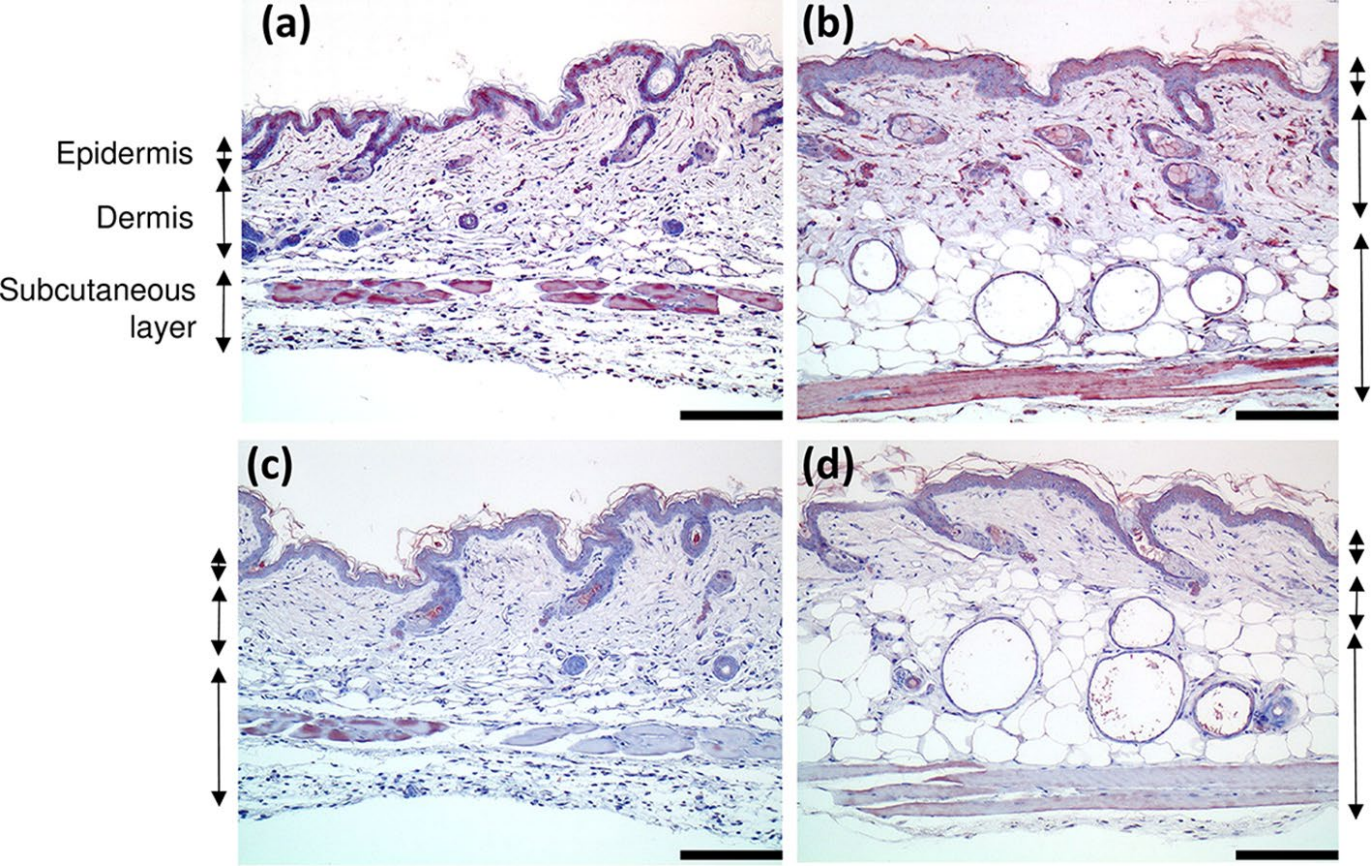
Representative histological images of immunostaining for taurine in the skin. The skin sections of 6-week-old (**a**) and 24-week-old mice (**b**) were stained with hematoxylin eosin (HE) and anti-taurine antibody. Taurine was stained red. Bar 300 µm. The corresponding negative control sections are shown in (**c**) and (**d**)

To assess the effect of aging on the localization of taurine in the skin, immunohistochemical analysis was carried out using anti-taurine antibody. In young mice, mononuclear infiltration of the dermis and subcutaneous tissues and atrophy of hair follicles were observed. On the other hand, in aged mice, cysts were formed in the hair follicle, and granulation was observed around the cysts. Immunoreactivity for taurine was observed in the upper part of the spinous layer, in atrophic hair follicles, in the mesenchymal cells under the dermis, and in the muscles in the young 6-week-old mice (Fig. 3a). A similar distribution profile of taurine immunoreactivity was also observed in the skin of the aged 24-week-old mice (Fig. 3b). Consistent with the quantitative assessment using HPLC, immunoreactivity for taurine in the skin was stronger in young animals as compared to that in their aged counterparts.

### Effect of taurine supplementation of drinking water

**Fig. 4:**
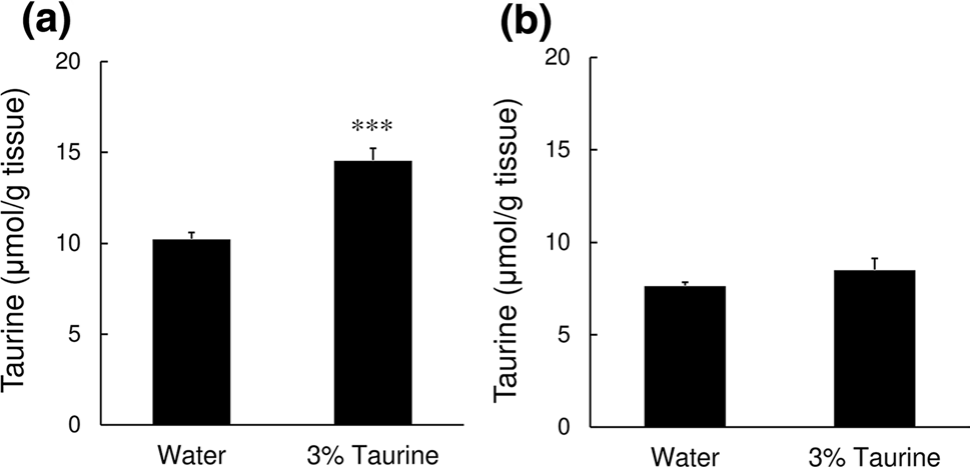
Effect of taurine supplementation of drinking water on the skin taurine content. Drinking water supplemented with 3% (w/v) taurine solution was administered ad libitum for 4 weeks to 35-week-old hairless mice, and the taurine contents in the epidermis (**a**) and dermis (**b**) of the skin of the back were determined by HPLC. Values represent the mean ± SE of 5 animals. The values were significantly different from those in the control mice (water), determined by Student’s *t* test. ***p < 0.001

We next examined the effect of taurine supplementation of the drinking water of the animals on the skin taurine content. Drinking water containing 3% taurine was given for 4 weeks to aged 35-week-old mice. The taurine supplementation significantly increased the taurine content in the epidermis (Fig. 4a), but not in the dermis (Fig. 4b).

## Discussion

The present study clearly demonstrated a decrease of the taurine content of the skin with age in both hairless mice and rats. In contrast, the taurine content of the sole remained unchanged with age. A few other studies have demonstrated an age-related decline of the tissue taurine contents. The taurine levels of the liver and kidney, as well as the serum taurine level, were decreased in aged male Fischer 344 rats, as compared to those in their adult counterparts, accompanied by a reduced urinary excretion of taurine (Dawson et al. 1996; Eppler and Dawson 1998). Age-related decline of urinary taurine excretion was also reported in aged female Wistar rats, probably as a result of renal conservation of taurine (Corman et al. 1985). The reduced taurine content of the liver with age appears to be associated, at least in part, with reduced taurine biosynthesis. The activities of the rate-limiting enzymes of taurine synthesis, that is, of cysteine sulfonate decarboxylase (CSD) and cysteine dioxygenase (CDO), have been shown to be reduced in the livers of aged Fischer 344 rats (Eppler and Dawson 1999). The present finding that oral taurine supplementation significantly increased the epidermal taurine content suggests that taurine is supplied mainly through the blood stream to the skin.

The present study confirmed that the skin taurine content decreased with age. It is well established that aging affects the basal metabolic rate and reduces biologically active components, such as vitamin C (van der Loo et al. 2003), coenzyme Q10 (Barcelos and Haas 2019), and carnitine (Costell et al. 1989). The skin content of collagen was also decreased with age due to a reduction of collagen synthesis (Rittié and Fisher 2015). These age-related changes result in skin dysfunction and wrinkle formation. The present study indicated that epidermal taurine is mainly supplied via the blood vascular system, because the taurine supplementation could increase the taurine level in the epidermis. In the skin, age-related alterations in the normal skin condition, including impairment of the basal metabolism and cardiovascular function lead to a reduction in the vascular vessel-mediated supply of taurine to the skin. The reduced taurine supply due to aging may result in age-related taurine depletion in the skin. As taurine is thought to play more important roles as an osmolyte in the epidermis, than in the dermis, orally supplemented taurine may be preferentially supplied to the epidermis, in comparison to the dermis. This may be the reason why taurine treatment increased the taurine content in the epidermis, not the dermis.

Previous immunohistochemical analyses have shown that taurine was distributed in the keratinocytes of the granular and upper spinous layers of the epidermis in dogs and Wister rats (Lobo et al. 2001). Sebaceous and muscle cells of the dermis were also immunopositive. A microradioautographic study using radiolabeled taurine showed that a very high density of taurine was present in the epidermis. (Watanabe et al. 1995). Our study revealed that taurine was present in the epidermis, dermis, and muscle layer of hairless mice. A quantitative analysis using HPLC showed that the taurine content of the epidermis was almost the same as that of the dermis. Hairless mice suffer from exposure to relatively harsh external environments, such as UV irradiation and changes in moisture content and temperature due to their nonhairy characteristic. UV irradiation induces inflammation, including an increase in the production and secretion of pro-inflammatory mediators, not only in the epidermis but epidermis (Pillai et al. 2005). Taurine is known to improve these pathophysiological conditions. For example, taurine accumulates in dermal fibroblast and inhibits UVA-induced IL-6 mRNA expression (Warskulat et al. 2008). Thus, much more taurine may be needed to counteract dermal changes in hairless mice than common hairy mice. This may be why the distribution of taurine was found to differ in hairless mice as compared to common hairy mice. Although the skin taurine content was markedly decreased in aged animals as compared to their younger counterparts, no marked differences in the localization of taurine were observed between the two age groups.

The pivotal role of taurine in the epidermis has been documented before. Taurine acts as an important organic osmolyte required for keratinocyte hydration (Janeke et al. 2003). When human skin is exposed to a dry environment, an increase in transepidermal water loss (TEWL) is induced, accompanied by the synthesis of barrier lipids and accumulation of taurine in the outermost granular keratinocyte layer. Topical application of taurine has been shown to decrease the TEWL observed after repeated exposure to surfactant in reconstructed epidermis (Anderheggen et al. 2006). Keratinocytes express the Na^+^- and Cl^−^-dependent, high-affinity taurine transporter (Grafe et al. 2004). The localization of taurine in the skin is well correlated with the expression of the taurine transporter in human keratinocytes—higher expression in the epidermis and lower expression in the dermis (Janeke et al. 2003). In cultured human keratinocytes, taurine accumulation has been shown to occur in an osmolarity-dependent manner, accompanied by increased mRNA levels of the taurine transporter (Janeke et al. 2003). An in vivo study using taurine transporter knockout mice showed that epidermal taurine is involved in the photoprotection of skin (Rockel et al. 2007). Recently, the study using human skin samples showed that epidermal keratinocytes possess osmolyte-mediated cell volume regulatory mechanisms, which is compromised in aging (Foster et al. 2020). Furthermore, taurine has been shown to enhance the structure and function of tight junction in keratinocytes (El-Chami et al. 2020). Thus, taurine is important for protection of the epidermal barrier.

**Fig. 5:**
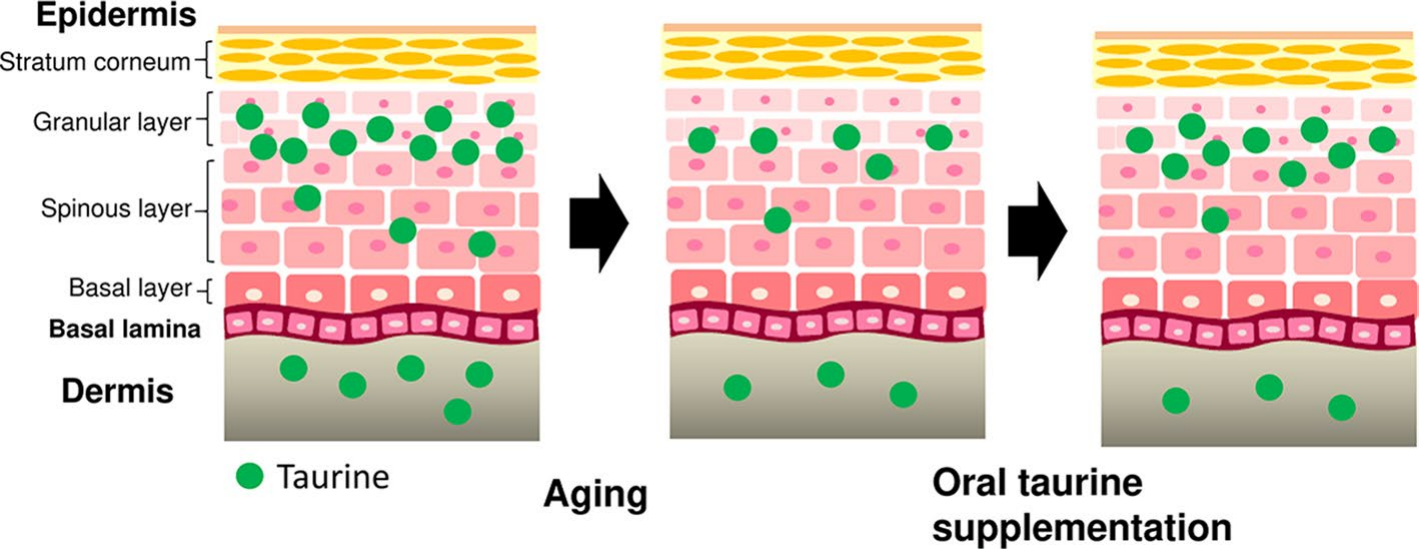
Schematic diagram showing the age-related decline in the taurine content in the skin and the effect of oral taurine supplementation

A schematic diagram of the age-related changes in the taurine content is shown in Fig. 5. Taurine is present in the epidermis and dermis. Taurine plays an important role as an organic osmolyte especially in the epidermis in maintaining homeostasis of the skin, including hydration of the skin. Aging causes taurine depletion in the skin, while oral supplementation with taurine restores the decreased taurine content in the epidermis.

In conclusion, the present study revealed that the skin taurine content decreases with age, similarly to other biologically active components. The decline in biological components, including taurine, may contribute to impaired skin hydration and wrinkle formation in aged skin. These findings also suggest an important role of taurine in maintaining a normal skin function, especially in the epidermis. Furthermore, the findings that oral taurine supplementation could increase the epidermal taurine content raise the possibility that oral treatment with taurine may be effective in the prevention of age-related deterioration in the skin. Further studies are needed to clarify the age-related changes and establish the beneficial roles of taurine in human skin.

## References

[CR1] Al-Nuaimi Y, Sherratt MJ, Griffiths CE (2014). Skin health in older age. Maturitas.

[CR2] Anderheggen B, Jassoy C, Waldmann-Laue M, Förster T, Wadle A, Doering T (2006). Taurine improves epidermal barrier properties stressed by surfactants-a role for osmolytes in barrier homeostasis. J Cosmet Sci.

[CR3] Barcelos IP, Haas RH (2019). CoQ10 and aging. Biology (Basel).

[CR4] Corman B, Pratz J, Poujeol P (1985). Changes in anatomy, glomerular filtration, and solute excretion in aging rat kidney. Am J Physiol.

[CR5] Costell M, O’Conner JE, Grisolia S (1989). Age-dependent decrease of carnitine content in muscle of mice and humans. Biochem Biophys Res Commun.

[CR6] Dawson R, Eppler B, Patterson TA, Shih D, Liu S (1996). The effects of taurine in a rodent model of aging. Adv Exp Med Biol.

[CR7] Dawson R, Biasetti M, Messina S, Dominy J (2002). The cytoprotective role of taurine in exercise- induced muscle injury. Amino Acids.

[CR8] El-Chami C, Foster AR, Johnson C, Clausen RP, Cornwell P, Haslam IS, Steward MC, Watson REB, Young HS, O'Neill CA (2020) Organic osmolytes increase expression of specific tight junction proteins in skin and alter barrier function in keratinocytes. Br J Dermatol 10.1111/bjd.19162 32348549

[CR9] Eppler B, Dawson R (1998). The effects of aging on taurine content and biosynthesis in different strains of rats. Adv Exp Med Biol.

[CR10] Eppler B, Dawson R (1999). Cysteine sulfinate decarboxylase and cysteine dioxygenase activities do not correlate with strain-specific changes in hepatic and cerebellar taurine content in aged rats. Mech Ageing Dev.

[CR11] Foster AR, El Chami C, O'Neill CA, Watson REB (2020). Osmolyte transporter expression is reduced in photoaged human skin: implications for skin hydration in aging. Aging Cell.

[CR12] Grafe F, Wohlrab W, Neubert RH, Brandsch M (2004). Functional characterization of sodium- and chloride-dependent taurine transport in human keratinocytes. Eur J Pharm Biopharm.

[CR13] Huxtable RJ (1992). Physiological actions of taurine. Physiol Rev.

[CR14] Ideishi M, Miura S, Sakai T, Sasaguri M, Misumi Y, Arakawa K (1994). Taurine amplifies renal kallikrein and prevents salt-induced hypertension in Dahl rats. J Hypertens.

[CR15] Ito T, Kimura Y, Uozumi Y, Takai M, Muraoka S, Matsuda T, Ueki K, Yoshiyama M, Ikawa M, Okabe M, Schaffer SW, Fujio Y, Azuma J (2008). Taurine depletion caused by knocking out the taurine transporter gene leads to cardiomyopathy with cardiac atrophy. J Mol Cell Cardiol.

[CR16] Ito T, Yoshikawa N, Inui T, Miyazaki N, Schaffer SW, Azuma J (2014). Tissue depletion of taurine accelerates skeletal muscle senescence and leads to early death in mice. PLoS ONE.

[CR17] Ito T, Yoshikawa N, Ito H, Schaffer SW (2015). Impact of taurine depletion on glucose control and insulin secretion in mice. J Pharmacol Sci.

[CR18] Janeke G, Siefken W, Carstensen S, Springmann G, Bleck O, Steinhart H, Höger P, Wittern KP, Wenck H, Stäb F, Sauermann G, Schreiner V, Doering T (2003). Role of taurine accumulation in keratinocyte hydration. J Invest Dermatol.

[CR19] Lobo MV, Alonso FJ, Latorre A, del Río RM (2001). Immunohistochemical localization of taurine in the rat ovary, oviduct, and uterus. J Histochem Cytochem.

[CR20] Madison KC (2003). Barrier function of the skin: "la raison d'être" of the epidermis. J Invest Dermatol.

[CR21] Pillai S, Oresajo C, Hayward J (2005). Ultraviolet radiation and skin aging: roles of reactive oxygen species, inflammation and protease activation, and strategies for prevention of inflammation-induced matrix degradation—a review. Int J Cosmet Sci.

[CR22] Rittié L, Fisher GJ (2015). Natural and sun-induced aging of human skin. Cold Spring Harb Perspect Med.

[CR23] Rockel N, Esser C, Grether-Beck S, Warskulat U, Flögel U, Schwarz A, Schwarz T, Yarosh D, Häussinger D, Krutmann J (2007). The osmolyte taurine protects against ultraviolet B radiation-induced immunosuppression. J Immunol.

[CR24] Schaffer S, Takahashi K, Azuma J (2000). Role of osmoregulation in the actions of taurine. Amino Acids.

[CR25] Schaffer S, Azuma J, Takahashi K, Mozaffari M (2003). Why is taurine cytoprotective?. Adv Exp Med Biol.

[CR26] Sergeeva OA, Fleischer W, Chepkova AN, Warskulat U, Häussinger D, Siebler M, Haas HL (2007). GABAA-receptor modification in taurine transporter knockout mice causes striatal disinhibition. J Physiol.

[CR27] van der Loo B, Bachschmid M, Spitzer V, Brey L, Ullrich V, Lüscher TF (2003). Decreased plasma and tissue levels of vitamin C in a rat model of aging: implications for antioxidative defense. Biochem Biophys Res Commun.

[CR28] Warskulat U, Borsch E, Reinehr R, Heller-Stilb B, Mönnighoff I, Buchczyk D, Donner M, Flögel U, Kappert G, Soboll S, Beer S, Pfeffer K, Marschall HU, Gabrielsen M, Amiry-Moghaddam M, Ottersen OP, Dienes HP, Häussinger D (2006). Chronic liver disease is triggered by taurine transporter knockout in the mouse. FASEB J.

[CR29] Warskulat U, Brookmann S, Felsner I, Brenden H, Grether-Beck S, Häussinger D (2008). Ultraviolet A induces transport of compatible organic osmolytes in human dermal fibroblasts. Exp Dermatol.

[CR30] Watanabe H, Watanabe M, Jo N, Kiyokane K, Shimada M (1995). Distribution of [1,2-3H]taurine in the skin of adult and newborn mice studied by microradioautography. Cell Mol Biol (Noisy-le-grand).

